# Supplementary material for: RADAR: differential analysis of MeRIP-seq data with a random effect model
Source: Genome Biol. 2019 Dec 23;20:294. doi: 10.1186/s13059-019-1915-9 (PMC6927177; doi:10.1186/s13059-019-1915-9)
Supplement: Supplementary file 1 — Additional file 1: Figure S1. Scatter plot of read count. Figure S2. Read count distribution of INPUT data. Figure S3. Sequencing depth distribution of m6A-seq in published literatures. Figure S4. Variability distribution comparing RNA-seq and m6A-seq (MeRIP-seq). Figure S5. Evaluating performances of benchmarked methods on simulated data using sliding thresholds. Figure S6. P-value and effect size estimates on simulated data. Figure S7. Coverage plot of individual samples for example DM sites and bogus sites in the T2D dataset. Figure S8. Compare the methods to adjust for gene expression level. Figure S9. Compare results obtained from shallower sequence depth with that from original depth. Figure S10. Motif analysis and topological distribution of putative DM sites. Figure S11. Coverage plot to visualize differential m6A peaks in ovarian cancer. Figure S12. Representation of Insulin/IGF1-AKT-PDX1 pathway. Figure S13. Analysis of statistical power and the number of replicates. Table S1. Enriched DM genes in KEGG pathways in the T2D dataset. Table S2. Selected DM sites for experimental validation. Table S3. Oligo probes sequences and qPCR primer sequences. [file 13059_2019_1915_MOESM1_ESM.pdf]

Additional file 1

RADAR: Differential analysis of MeRIP-seq data with a random effect model

Zijie Zhang[2,3], Qi Zhan[1], Mark Eckert[4], Allen Zhu[2,3], Agnieszka Chryplewicz[4], Dario F De Jesus[5], Decheng Ren[6], Rohit N Kulkarni[5], Ernst Lengyel[4], Chuan He[2,3], Mengjie Chen[1].

[1]Section of Genetic Medicine, Department of Medicine, Department of Human Genetics, The University of Chicago, Chicago, IL 60637, USA;

[2]Department of Chemistry, Department of Biochemistry and Molecular Biology, and Institute for Biophysical Dynamics, The University of Chicago, Chicago, IL 60637, USA;

[3]Howard Hughes Medical Institute, The University of Chicago, Chicago, IL 60637, USA;

[4]Department of Obstetrics & Gynecology, Section of Gynecologic Oncology, The University of Chicago, Chicago, IL 60637, USA;

[5]Section of Islet Cell and Regenerative Biology, Joslin Diabetes Center and Harvard Medical School, Boston, MA 02215 USA;

[6] Section of Endocrinology, Diabetes & Metabolism, Department of Medicine, The University of Chicago, Chicago, IL 60637 USA.

Correspondence to: mengjiechen@uchicago.edu & chuanhe@uchicago.edu

Fig. S1

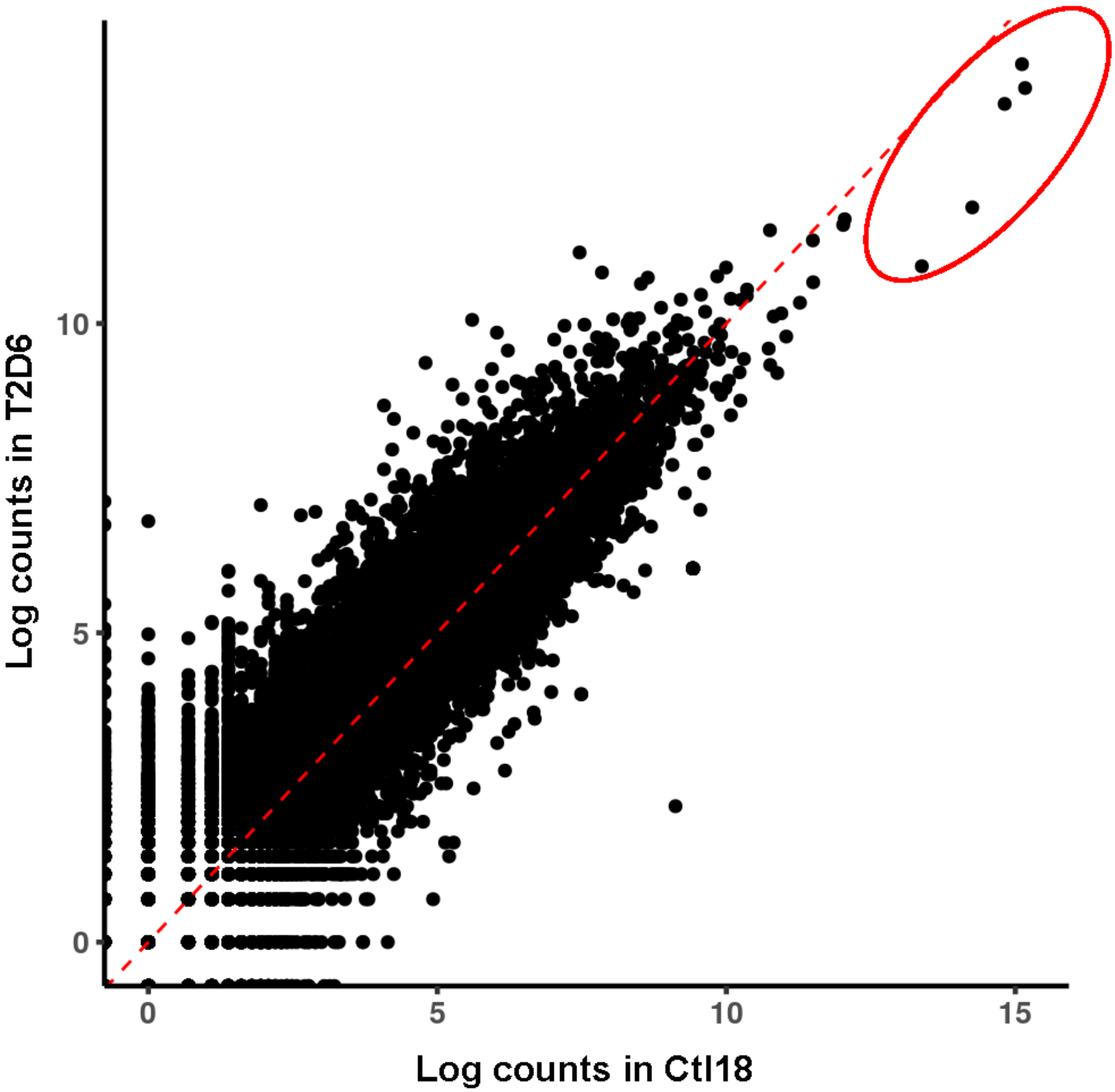

Fig. S1. Scatter plot of read count. The input library log read counts of a sample from one experimental group are plotted against a sample from another experimental group. Shown is an example scenario where highly expressed genes can result in underestimation of other genes when normalizing by total coverage. The highly expressed genes that can strongly influence the scaling factor estimation are highlighted by red circles.

Fig. S2

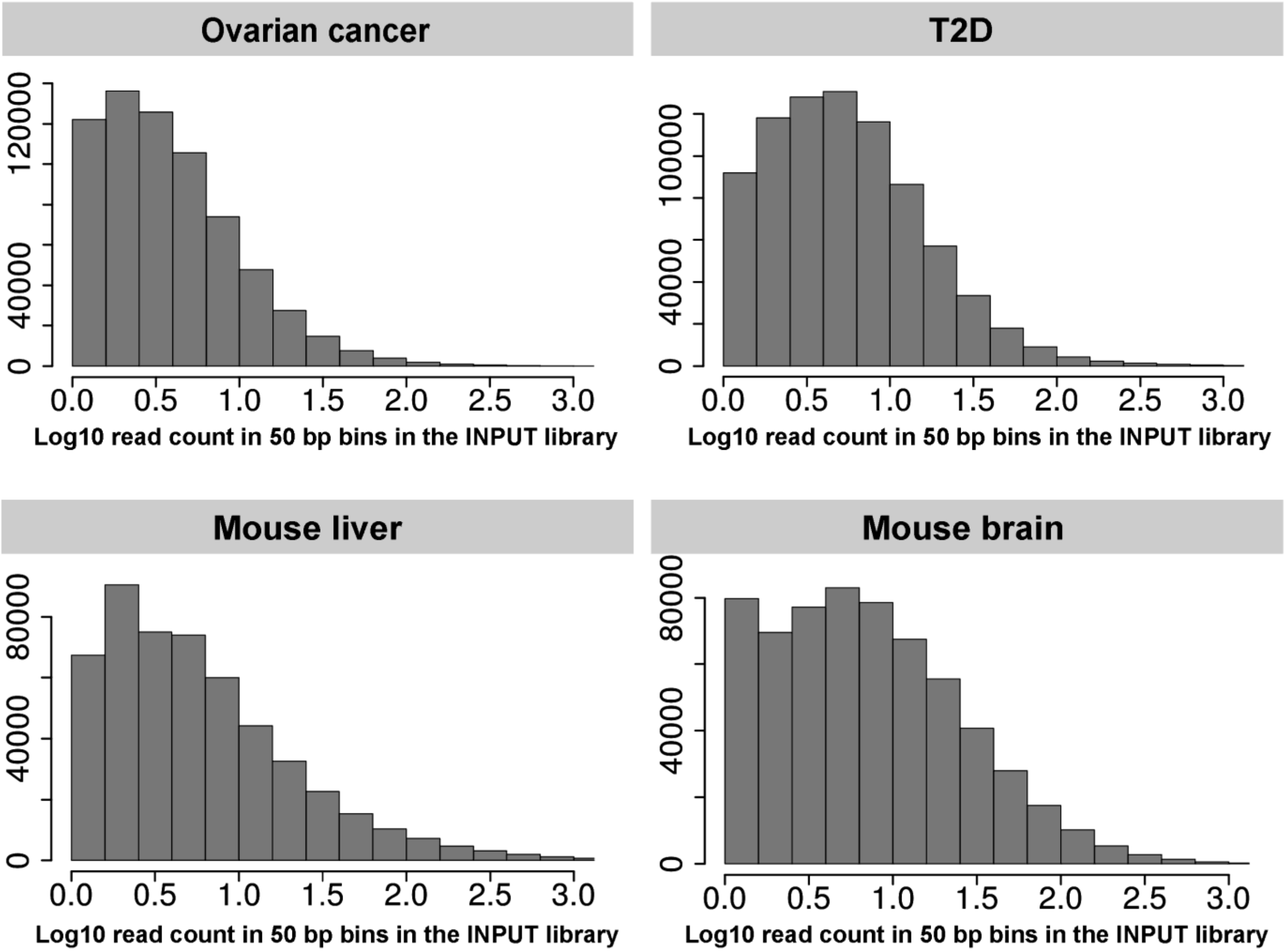

Fig. S2. Read count distribution of INPUT data. Distribution of read count c_i (Fig. 1A) in a 50bp bin of input library from real m_6A-seq datasets. The read count is shown in log10 scale.

Fig. S3

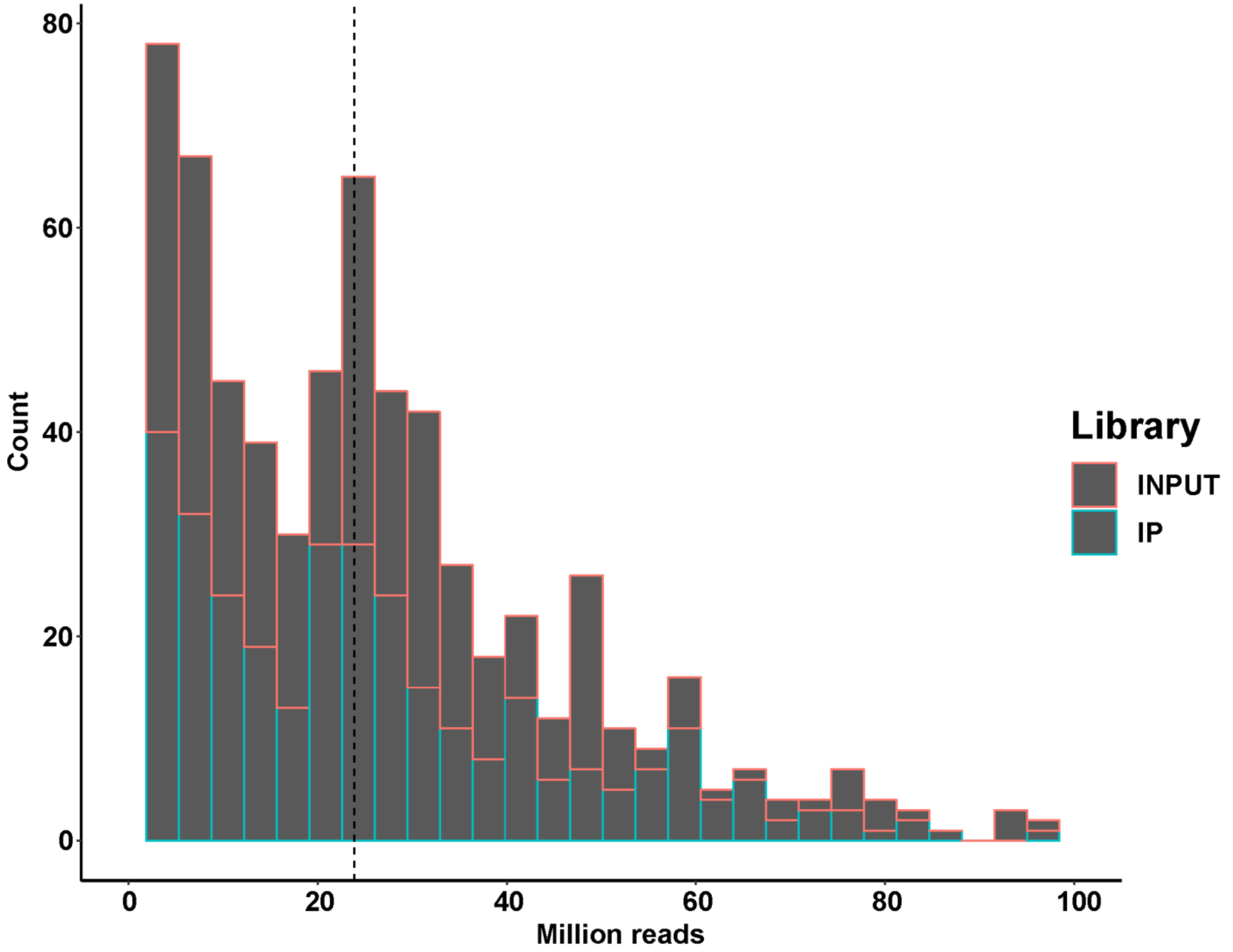

Fig. S3. Sequencing depth distribution of m6A-seq in published literatures. Distribution of sequencing depth (million reads) drawn form a m6A-seq database (unpublished observations) is shown by histogram. The database included 339 datasets from published literatures.

Fig. S4

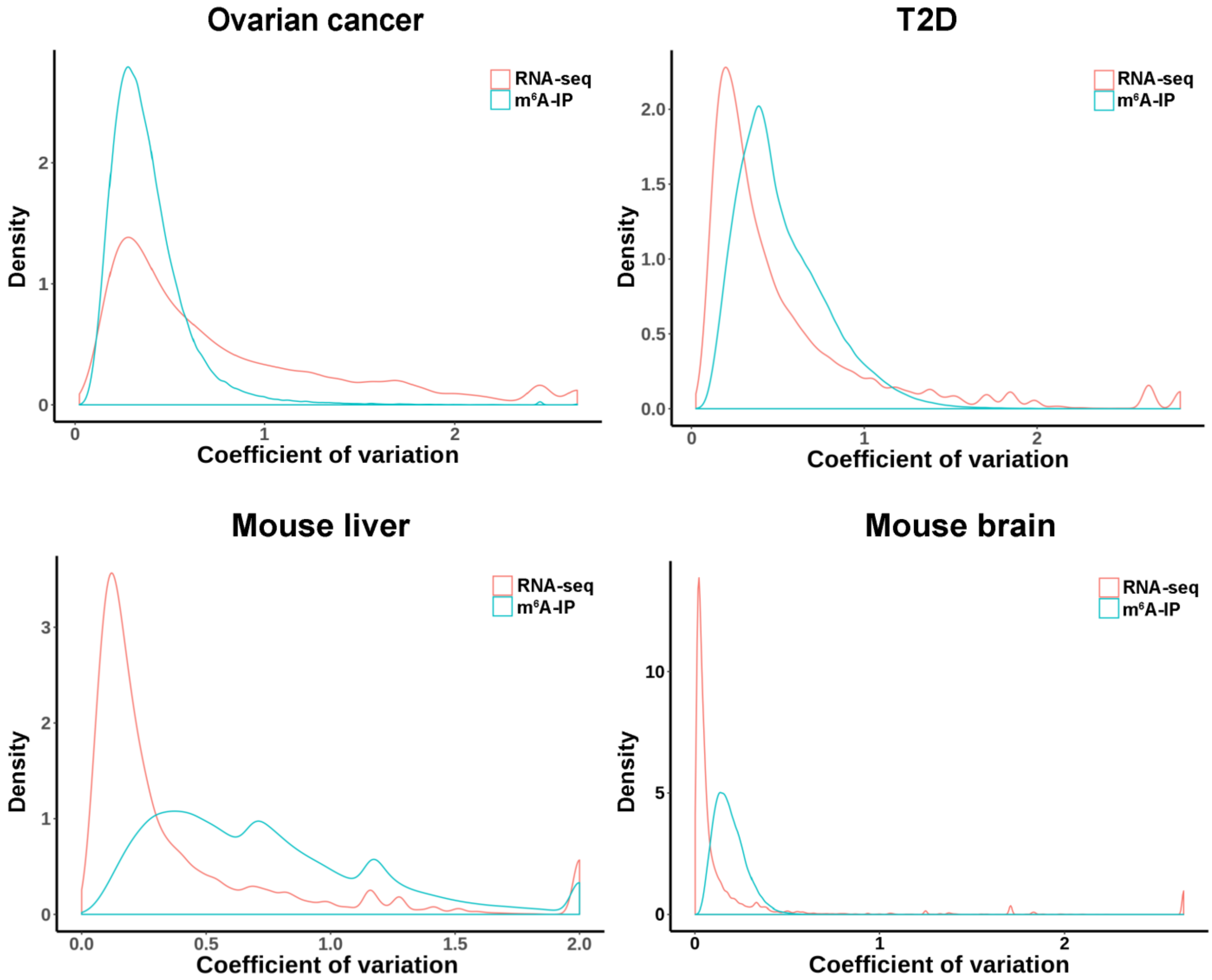

Fig. S4. Variability distribution comparing RNA-seq and m6A-seq (MeRIP-seq). Density plot comparing variabilities of m6A-seq data with RNA-seq data. Variability is represented by coefficient of variation.

Fig. S5

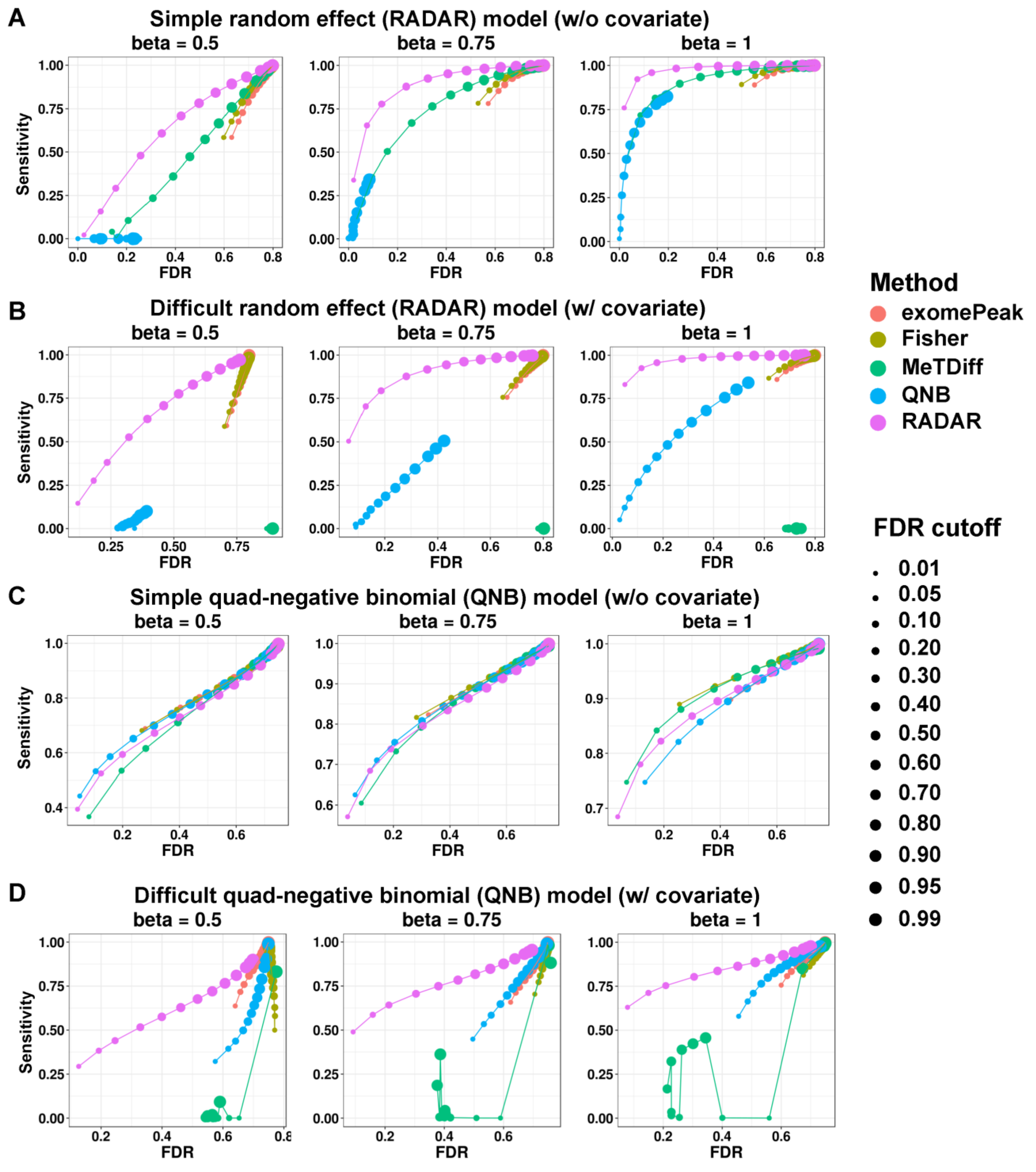

Fig. S5. Evaluating performances of benchmarked methods on simulated data using sliding thresholds. We evaluated the performance of RADAR and other methods by comparing the sensitivity and empirical FDR obtained by varying FDR threshold for selecting DM sites. The threshold of selecting DM sites are labeled by the size of data points.

Fig. S6

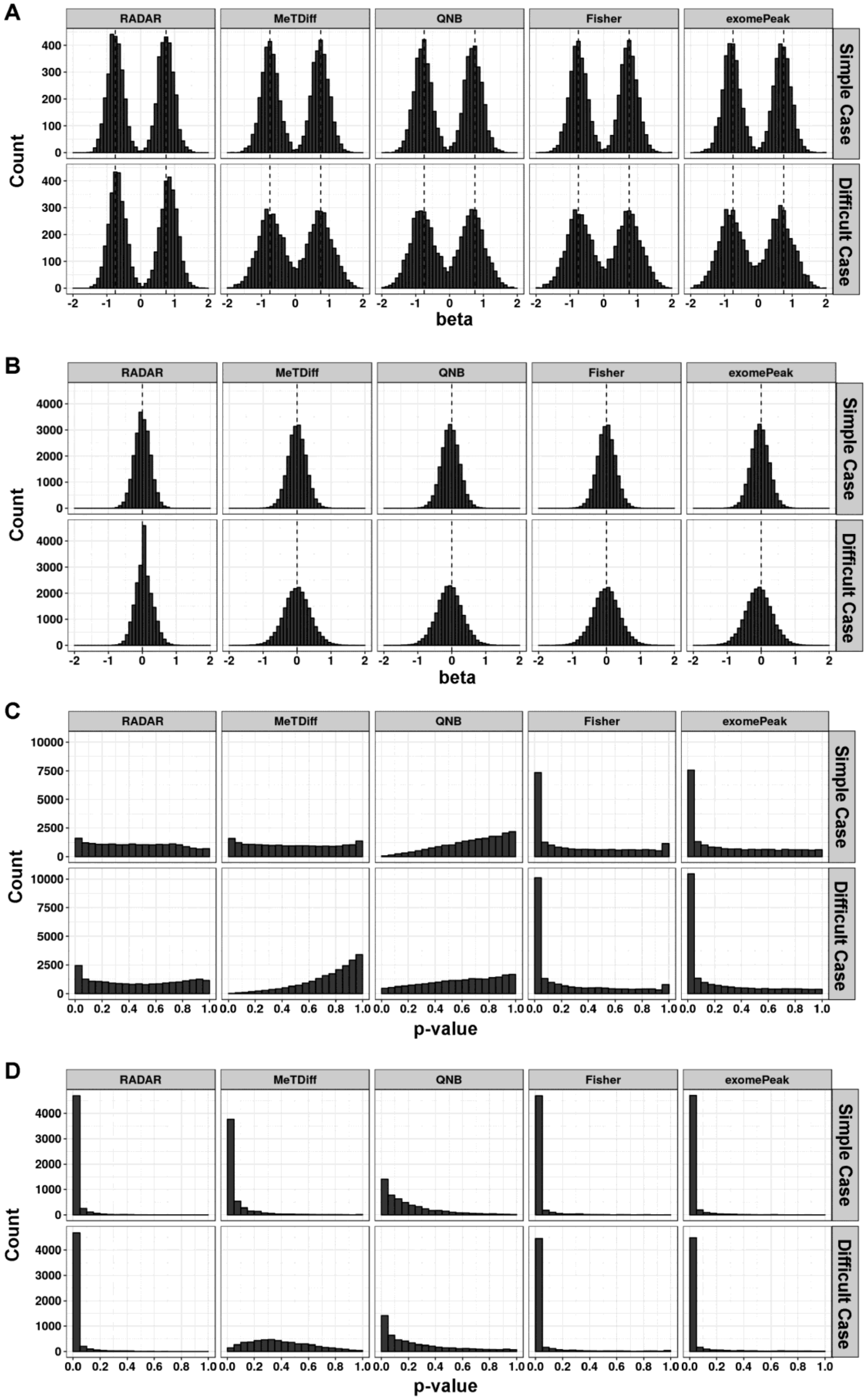

A
RADAR
MeTDiff
QNB
Fisher
exomePeak
Simple Case
Difficult Case
Count
beta
B
C
p-value
D

Fig. S6 – continued

Fig. S6. P-value and effect size estimates on simulated data. Using data simulated by random effect model of effect size = 0.75, we evaluated the precision of effect size and p-value estimates. (**a**) shows the distribution of effect size estimates in true differential sites and (**b**) shows the distribution of effect size estimates in Null sites where the true effect size is labeled by dashed line. (**c**) shows p-values distribution for Null sites where p-values are expected to be uniformly distributed. (**d**) shows p-values distribution for true differential sites where p-values are expected to be distributed near zero. In all panels, “simple case” refers to simulated data without covariates while “difficult case” refers to simulated data with a covariate.

Fig. S7

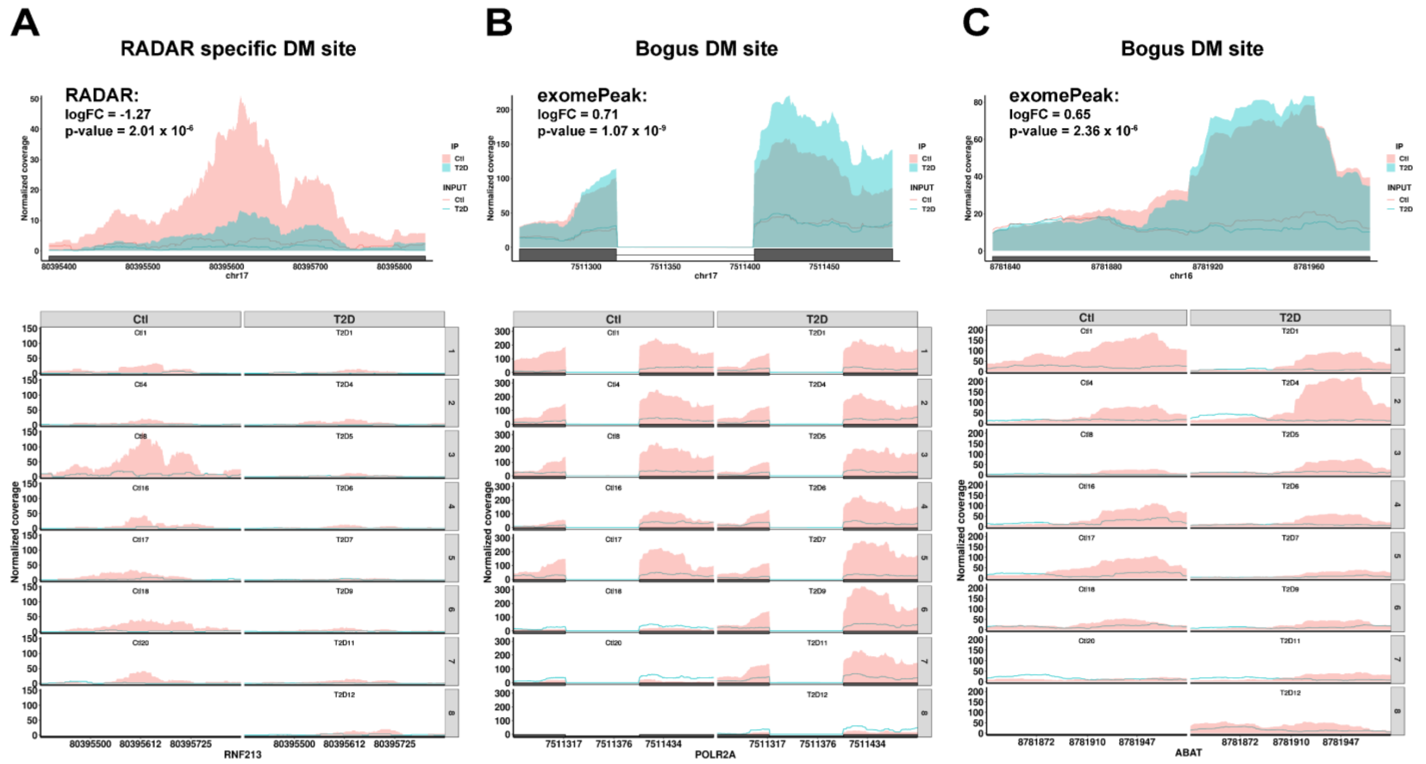

Fig. S7. Coverage plot of individual samples for example DM sites and bogus sites in the T2D dataset. We visualize raw data by showing coverage plot for three examples m_6A sites. (**a**) shows a putative DM site that was only detected by RADAR but missed by other methods. (**b**) shows a bogus DM site where difference between two groups was mainly driven by two strongly hypomethylated samples in the control group instead of consistent change among replicates. (**c**) shows another bogus DM site that were mainly driven by an outlier hypermethylated sample in the T2D samples.

Fig. S8

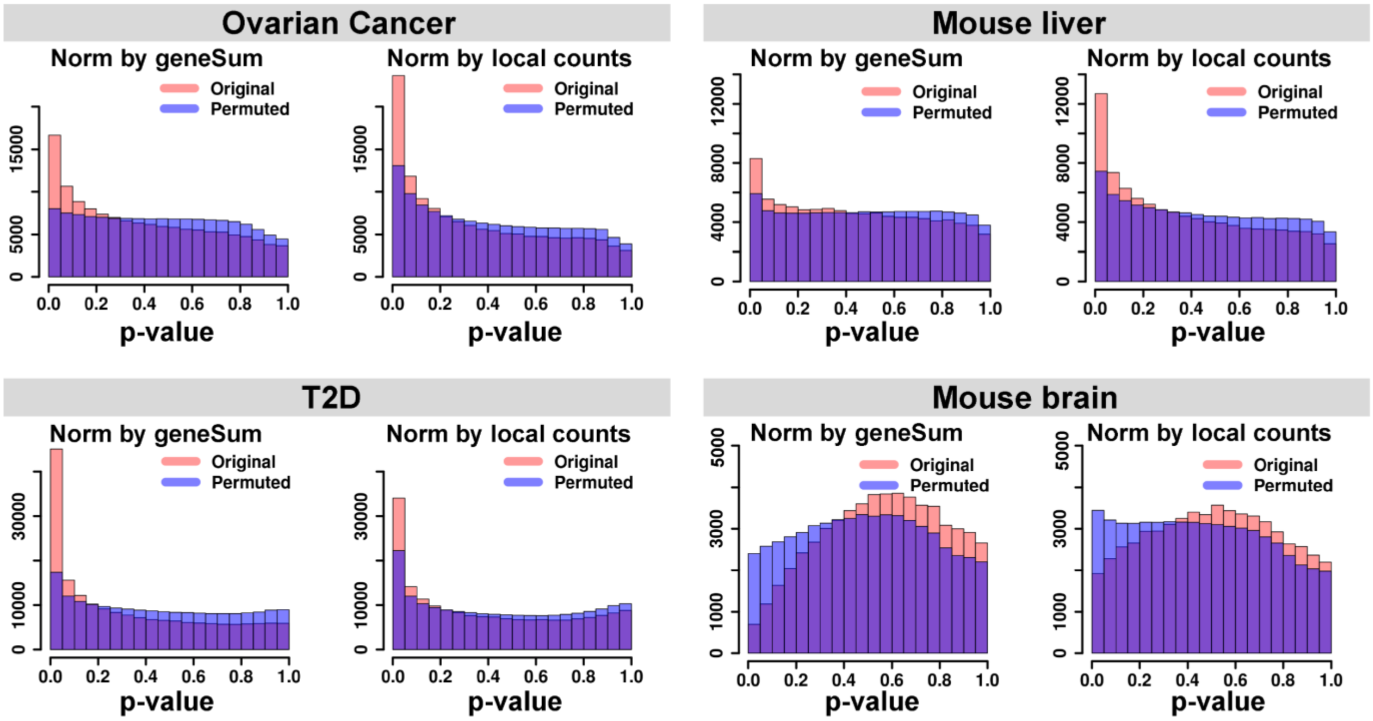

Fig. S8. Compare the methods to adjust for gene expression level. Local peak/bin read counts or gene level read counts of INPUT library can be used to account for pre-IP gene expression level variation. We compared the performance of two strategies to adjust for gene expression variation and showed the histogram of p-values from original tests and permutation tests.

Fig. S9

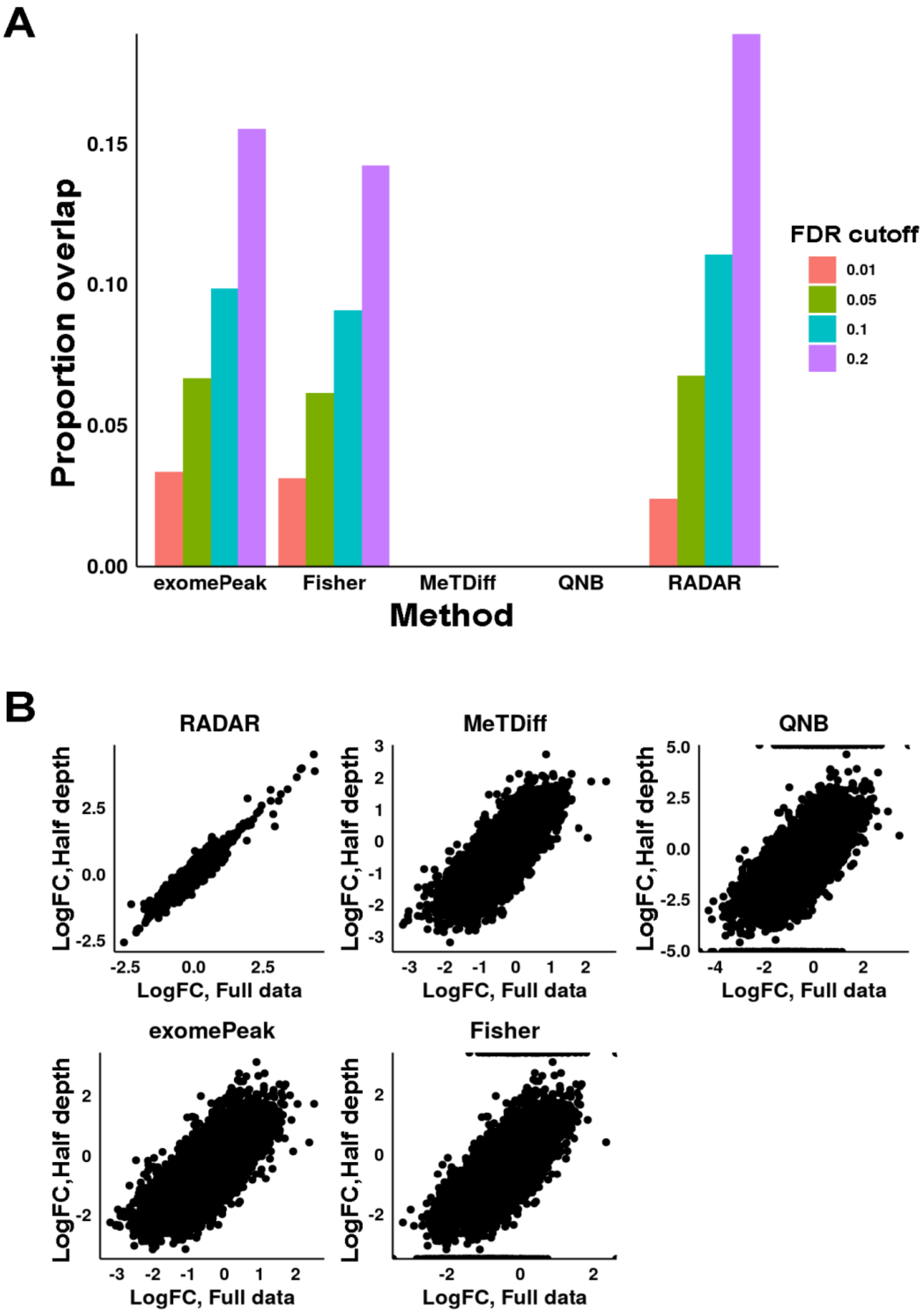

Fig. S9. Compare results obtained from shallower sequence depth with that from original depth. We sub-sampled sequence reads from INPUT libraries of the T2D dataset to obtain a dataset of shallower sequence depth (half of the original data). We applied the benchmarked methods to the sub-sampled data and compared the result with the result obtained from the original data. We show the proportion of sites positively identified in both datasets in (**a**) and plotted the estimated log fold changes against each other in (**b**).

Fig. S10

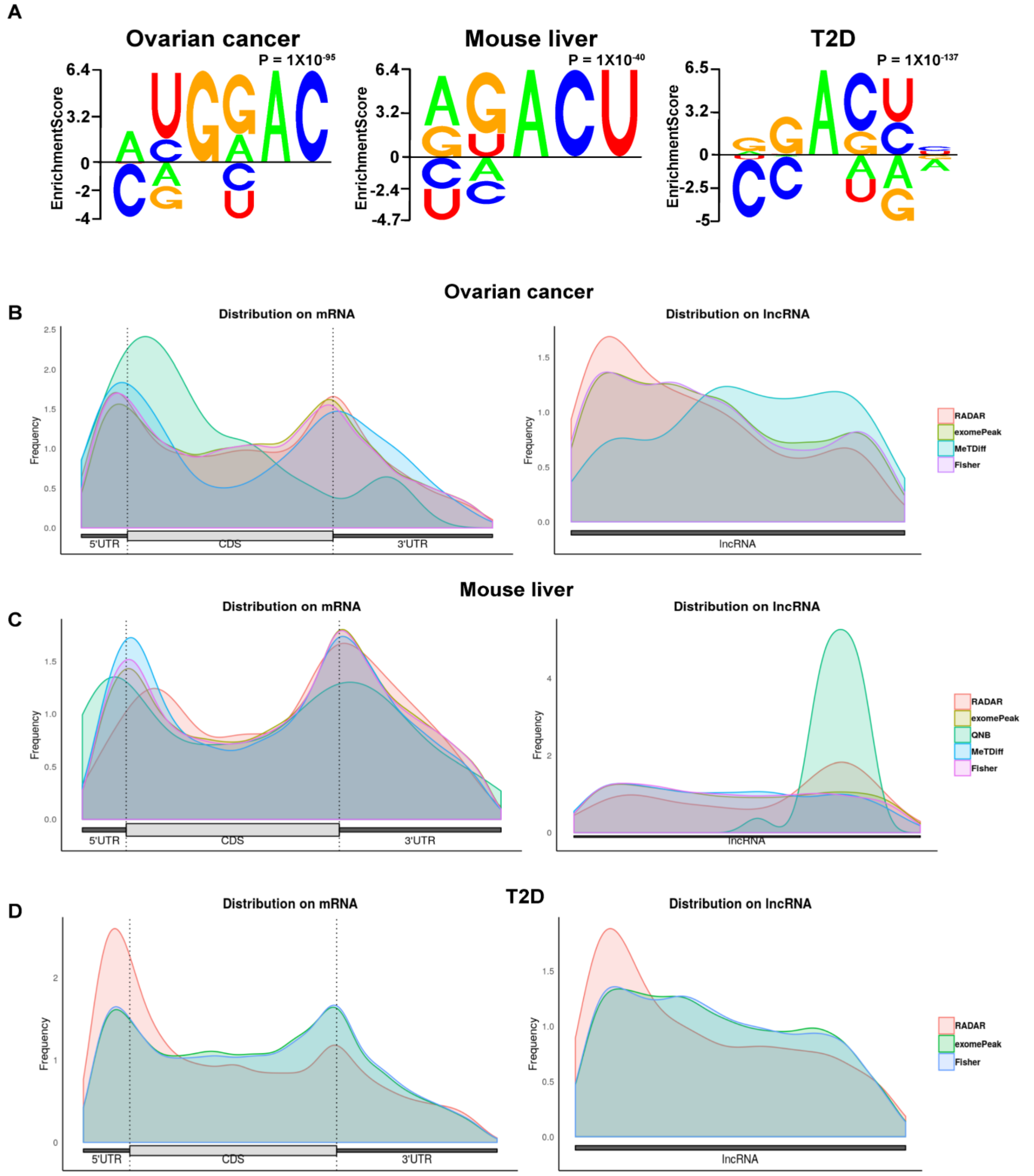

Fig. S10. Motif analysis and topological distribution of putative DM sites. We performed de-novo motif search analysis using Homer2 on the putative DM sites detected by RADAR on ovarian cancer, mouse liver and T2D datasets. (**a**) shows RADAR-detected DM sites were enriched for known m6A consensus motif—RRACU. (**b**) shows metagene plots of putative DM sites detected by different methods (method that detected too few DM sites in given dataset were not shown).

Fig. S11

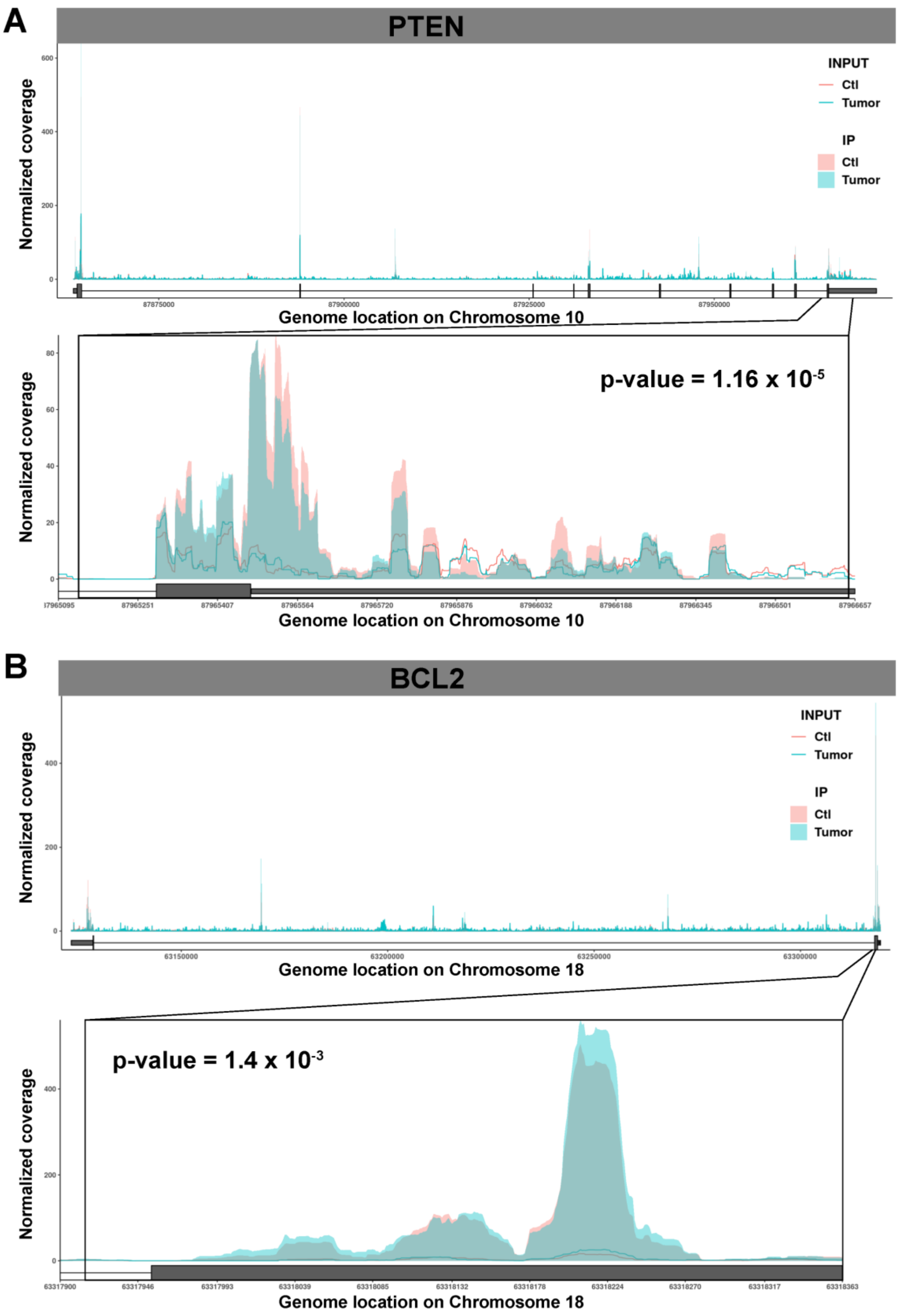

Fig. S11. Coverage plot to visualize differential m$_6$A peaks in ovarian cancer. Average coverage of each group is plotted for (**a**) PTEN and (**b**) BCL2. The coverages of both INPUT and IP are normalized by the expression level of target gene so that the coverages of IP samples are directly comparable.

Fig. S12

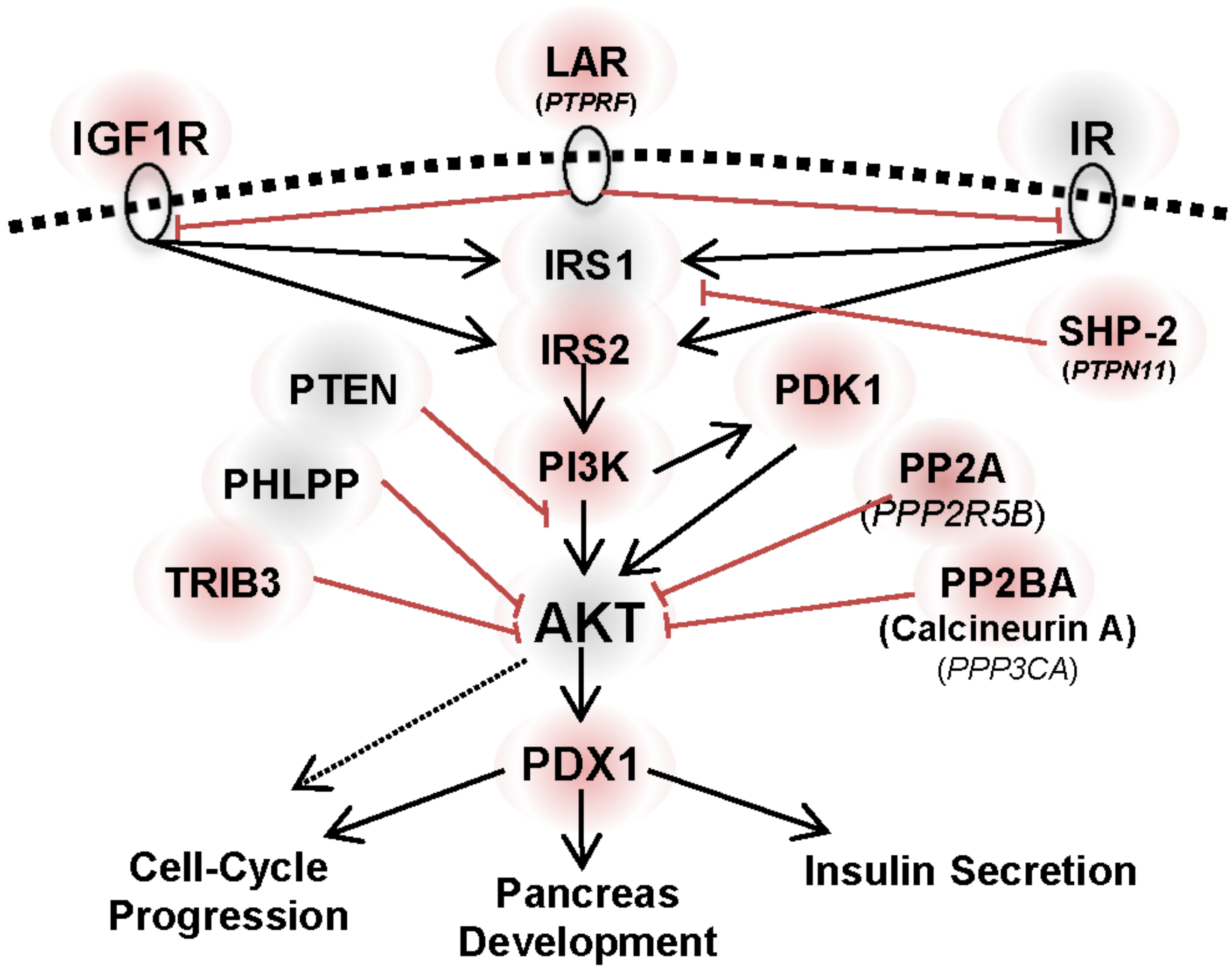

Fig. S12. Representation of Insulin/IGF1-AKT-PDX1 pathway. The diagram shows the Insulin/IGF1-AKT-PDX1 signaling pathway based on KEEG and Wikipathway annotations and depicts several m_6A hypomethylated genes (red shade) and unchanged genes (grey shade) in T2D as compared to Controls.

Fig. S13

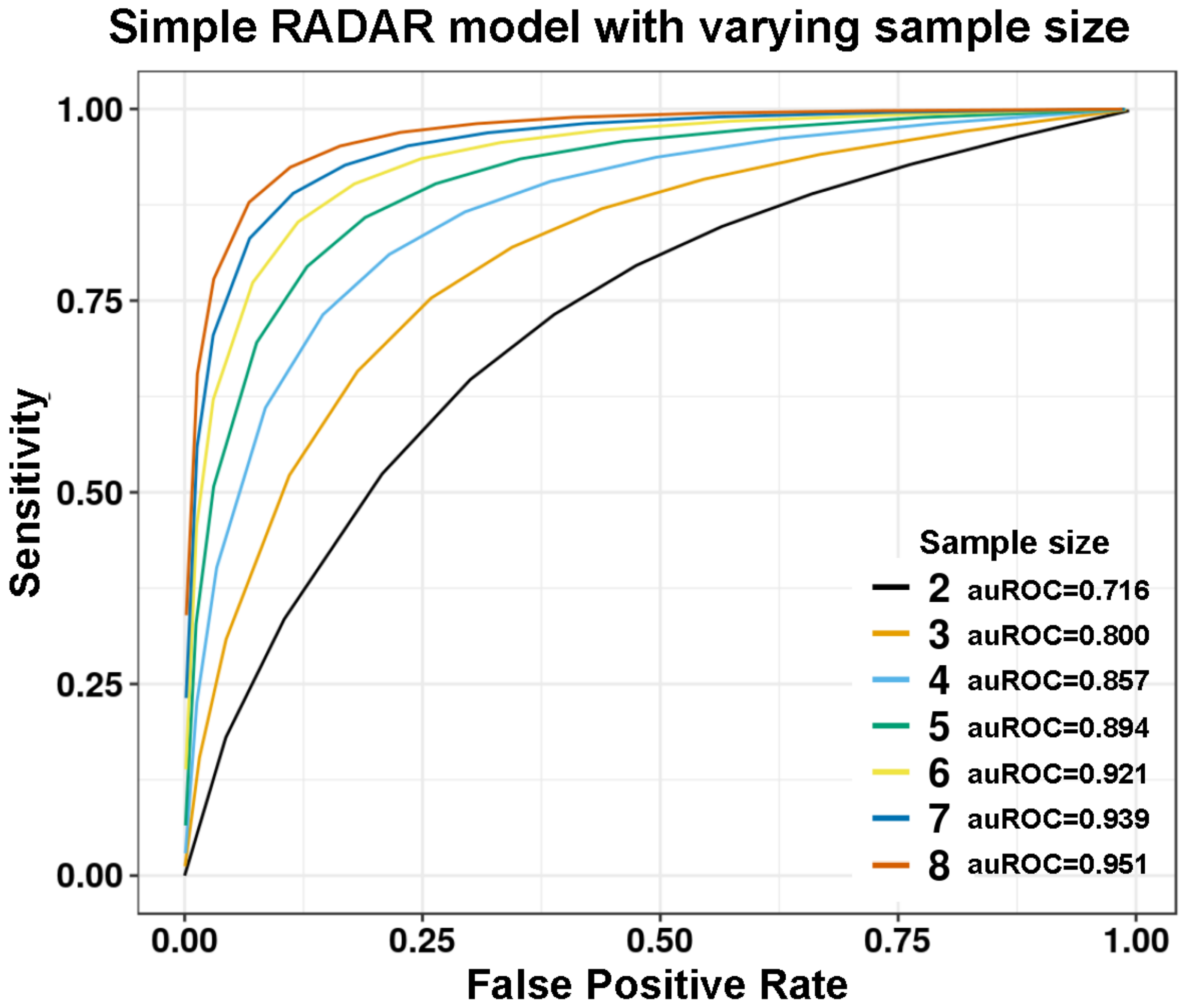

Fig. S13. Analysis of statistical power and the number of replicates. We plotted the sensitivity against the empirical FDR by varying the FDR cutoff for selecting predicted differential sites. The larger the area under the curve, the larger the power of the test.

Table S1

Pathway ID	Pathway Names	GeneRatio	BgRatio	pvalue	geneID (Entrez)	Count
hsa04510	Focal adhesion	62/1495	199/7878	2.08E-05	3678/3725/2909/1278/9475/2321/29780/10319/7094/3675/3480/3265/55742/7058/3672/5170/2889/10000/3693/394/5595/5500/2534/894/7057/5063/5296/1729/4233/595/1399/5290/2002/858/3910/3918/3915/1292/7422/5156/824/857/896/3694/54776/2932/5602/1284/103910/331/1282/3791/9564/208/2316/5829/2317/3655/5923/6714/81/4659	62
hsa04152	AMPK signaling pathway	41/1495	120/7878	5.25E-05	5526/6794/8408/1978/1080/3480/23417/5564/5170/10000/5528/84335/57521/2308/5209/2309/9586/8660/90993/5296/31/595/5290/3172/6885/10890/32/5518/51094/5529/3630/5862/5520/7248/5214/23411/6720/10488/208/5525/5527	41
hsa04928	Parathyroid hormone synthesis, secretion and action	37/1495	106/7878	7.18E-05	4325/3727/1958/4324/2260/4041/2770/5595/9368/9586/9138/6256/5144/2768/2771/90993/3710/11214/5567/2353/10672/846/6667/7421/1026/6257/9365/112/107/2767/4040/369/10488/4205/9935/5332/5566	37
hsa04150	mTOR signaling pathway	48/1495	153/7878	0.000144	6794/1856/8408/54361/1978/3480/3265/58528/7132/529/5170/10000/4041/84335/57521/79109/5595/64798/8321/55004/54541/96459/5296/5290/79726/90423/64121/9681/1857/83667/54468/84219/1975/3630/10325/23175/7248/4040/2932/3551/9470/389541/208/57600/9894/201163/8140/6520	48
hsa03015	mRNA surveillance pathway	32/1495	91/7878	0.000185	5526/7919/8106/26528/22985/5528/80335/5500/51585/65109/65110/100529063/23049/8189/9887/79869/53918/5518/5529/10250/5520/10482/140886/8761/5525/23293/5527/22794/1477/53981/10914/2935	32

hsa04933	AGE-RAGE signaling pathway in diabetic complications	34/1495	100/7878	0.000248	3725/1278/1958/3265/10000/4089/4088/2308/5595/7046/113026/183/1027/4790/5970/5296/1729/595/5290/7040/7422/6777/581/5590/5602/5580/5581/1284/1282/7042/208/51196/7056/5332	34
hsa04068	FoxO signaling pathway	42/1495	132/7878	0.000262	100132074/1387/6794/9455/9454/3480/3265/5564/5170/10000/4089/5934/4088/2308/5595/2309/7046/894/2033/1027/8660/5296/595/6789/5290/4193/7040/1026/3630/23411/1017/5602/3551/4303/1901/1454/7042/369/208/1032/1030/7874	42
hsa04530	Tight junction	51/1495	170/7878	0.000311	776/3725/6794/93643/9475/4771/1080/5962/23370/7122/5564/11346/4628/9368/9414/1740/8189/51421/9075/23327/595/56288/5567/4637/7082/1364/83700/100506658/5518/79784/10096/154796/84952/79778/5590/5520/64398/1365/5602/5581/1741/103910/4214/137075/8976/9223/8777/6714/81/5566/1739	51
hsa04010	MAPK signaling pathway	80/1495	295/7878	0.000314	3481/775/773/776/3725/6237/1843/3727/4215/6722/2321/9479/2260/3304/2005/3480/3265/1847/7132/8912/9448/100506012/9175/10000/5530/1850/5595/7046/5598/1435/3925/23162/3310/2768/4790/23542/5970/4233/5606/5567/6789/2353/3554/1399/22800/80824/2002/5494/7040/6885/4609/4803/7422/774/3303/5156/3630/7186/5602/781/3551/7042/8605/4137/369/3791/4214/208/3305/2316/51347/4775/7039/9261/2317/23118/9254/5923/57551/5566	80
hsa04950	Maturity onset diabetes of the young	13/1495	26/7878	0.000345	3651/389692/4821/6928/3087/6927/3170/3171/3172/3110/3630/168620/222546	13

hsa04071	Sphingolipid signaling pathway	38/1495	119/7878	0.000466	5526/9475/56848/3265/7132/4363/5170/10000/5528/2770/8877/5595/29956/2534/130367/2768/4790/2771/6609/5970/5296/10672/5290/5518/5529/581/5590/5520/7186/9846/5602/1901/5581/259230/208/5525/5527/5332	38
hsa04931	Insulin resistance	35/1495	108/7878	0.000567	7132/5564/5170/10000/51085/2308/5500/22877/8473/5781/9586/183/57761/11000/4790/8660/90993/5970/5296/5290/6945/32/3630/5590/5465/2932/5602/5580/3551/5581/6720/10488/208/5836/4792	35
hsa04520	Adherens junction	25/1495	72/7878	0.001119	1387/9855/2260/10163/3480/5777/4089/4088/5595/117178/7046/2534/2033/4233/56288/7082/10580/6885/8826/4008/999/8976/5797/6714/81	25
hsa04930	Type II diabetes mellitus	18/1495	46/7878	0.001147	775/773/776/3651/389692/5595/8660/5296/5290/774/3630/5590/5602/5580/80201/3551/5581/122809	18
hsa04919	Thyroid hormone signaling pathway	36/1495	119/7878	0.001924	1387/9862/10231/54361/7067/3265/5170/10000/488/2308/5595/113026/23389/6256/2033/6567/8202/5296/6548/595/5567/5290/4193/4609/6257/4855/9969/5214/2932/5469/208/51196/4853/5332/6714/5566	36
hsa04390	Hippo signaling pathway	44/1495	154/7878	0.002303	1856/9113/54361/4771/26524/7003/4089/4088/5500/7532/7046/8321/324/894/1740/595/55233/8313/56288/3689/656/23286/1857/7040/4609/7529/4092/5518/154796/8994/896/5590/5520/64398/2932/10413/999/1741/1454/7042/3398/84962/1490/1739	44
hsa03040	Spliceosome	38/1495	135/7878	0.005799	24148/7919/6625/1659/9785/3304/23020/10291/51362/23451/22985/84991/10929/494115/51729/3310/10713/988/10946/6100/9343/3303/343069/1655/9092/5093/4670/8175/10084/100534599/11325/57461/3305/144983/23350/58517	38

					/9879/26121	
hsa04210	Apoptosis	38/1495	136/7878	0.006614	2021/3725/3265/2081/7132/8837/5170/10000/823/153090/5595/1514/142/4790/3710/5970/5296/2353/8739/5290/1522/6709/4803/824/581/7186/5366/5602/3551/4170/331/143/208/1512/1519/4792/598/8772	38
hsa04066	HIF-1 signaling pathway	31/1495	109/7878	0.010223	4055/1387/2321/112399/7037/1978/817/3480/10000/5209/5595/2033/1027/4790/5970/5296/5290/5163/7422/3939/1026/3630/7076/5214/229/3162/80201/9470/112398/208/54583	31
hsa04392	Hippo signaling pathway - multiple species	11/1495	29/7878	0.013318	8642/9113/4771/26524/7003/55233/23286/8994/10413/1454/84962	11
hsa04024	cAMP signaling pathway	54/1495	214/7878	0.013347	775/776/1387/3725/6237/5443/9475/817/1080/5727/10000/2770/488/5595/5500/9586/2696/116/2149/2033/5139/5144/4790/2771/90993/5970/5296/6548/1908/5567/2353/2740/5290/22800/6662/6752/84152/112/135/64411/1909/2891/5465/107/5602/10488/2693/208/6751/51196/153/4792/5566/4659	54
hsa04151	PI3K-Akt signaling pathway	84/1495	354/7878	0.013348	3481/5526/3678/6794/1278/2321/2260/1978/3566/10319/3675/3480/3265/3326/2791/118788/7058/3672/5170/10000/5528/3693/5934/57521/5595/5585/2309/7532/9586/894/1435/54541/6256/2149/7057/1027/4790/90993/9170/5970/5296/4233/595/5290/4193/3910/3918/3915/1292/4609/4803/7422/7529/1026/5518/7184/4515/1975/5529/5156/3630/896/3694/5520/7248/1017/2932/3551/9470/4170/1284/5586/1282/3791/10488/208/5525/9223/5527/2790/7039/23239/598/3655	84

hsa04142	Lysosome	33/1495	123/7878	0.019934	4669/967/1203/8218/3482/4864/3425/1514/9583/8120/2990/6609/23163/10312/1522/6448/79158/410/2548/8907/8943/256471/55353/84572/1212/10947/162/8546/1512/3074/1519/23431/23062	33
hsa04120	Ubiquitin mediated proteolysis	36/1495	137/7878	0.021349	55585/63893/7327/22954/23295/27338/10075/23221/9320/51343/64750/9690/54926/89910/8451/23327/57448/51366/23759/4193/7322/5371/997/9246/7321/65264/8924/331/7326/4214/92912/11060/55958/4281/9616/51433	36
hsa04910	Insulin signaling pathway	36/1495	137/7878	0.021349	1978/3265/5261/5564/5170/2889/10000/57521/2308/5595/5500/8660/5296/31/5567/1399/5290/10580/2002/32/3630/5576/5590/7248/3636/2932/5602/80201/3551/9470/6720/122809/369/208/5836/5566	36
hsa04310	Wnt signaling pathway	41/1495	160/7878	0.022279	1387/3725/5176/1856/9475/54361/817/59343/1487/4041/5530/4089/4088/6425/8321/324/894/23002/2033/595/8313/5567/166336/1857/4920/6885/4609/5467/6423/80319/896/27130/4040/2932/5602/1454/8061/85409/4775/5332/5566	41
hsa01522	Endocrine resistance	27/1495	98/7878	0.023827	3725/3480/3265/10000/1031/5595/1027/8202/5296/595/5567/2353/5290/4193/6667/1026/4855/112/581/107/5602/369/5469/208/4853/6714/5566	27
hsa04330	Notch signaling pathway	15/1495	48/7878	0.028337	1387/9612/1856/1487/2033/55534/1840/113878/1857/4855/5664/6868/9794/151636/4853	15
hsa05202	Transcriptional misregulation in cancer	46/1495	186/7878	0.02959	84444/2321/2005/3480/466/51274/905/5914/1031/2130/6935/2308/4297/1025/6692/8148/894/3087/6256/1027/4790/221037/5970/9915/4233/4299/4221/4193/5546/904/6667/4609/4094/1026/6257/5371/8938/581/8861/1655/5327/33	46

					98/3486/648/598/6929	
hsa04927	Cortisol synthesis and secretion	19/1495	65/7878	0.029637	775/776/5443/8912/3777/5151/9586/183/90993/3710/5567/6667/112/107/2767/949/10488/5332/5566	19
hsa04371	Apelin signaling pathway	35/1495	137/7878	0.0342	6237/1958/56848/3265/4899/2791/5564/10000/4089/2770/8877/4088/5595/7046/30849/2771/3710/6548/595/5567/10672/22800/5289/112/6543/107/5327/999/5581/208/1490/4205/2790/5332/5566	35
hsa04722	Neurotrophin signaling pathway	31/1495	119/7878	0.034682	3725/4215/817/3265/5170/2889/10000/5595/2309/5781/5598/4790/5970/5296/1399/5290/4803/10782/5664/581/25/10818/2932/5602/5580/3551/4214/208/25970/9261/4792	31
hsa04512	ECM-receptor interaction	24/1495	88/7878	0.035636	3678/1278/2812/10319/3675/9899/1605/7058/3672/3693/960/9900/7057/3910/3918/3915/1292/6382/255743/3694/1284/1282/375790/3655	24
hsa04611	Platelet activation	32/1495	124/7878	0.036476	2909/1278/9475/2812/7094/10000/2770/5595/5500/2534/23365/9138/2149/2771/3710/5296/5567/10672/9002/5290/6786/112/5590/107/5739/103910/8605/208/5332/6714/5566/4659	32
hsa04915	Estrogen signaling pathway	35/1495	138/7878	0.037743	3725/5443/3304/3265/3326/2775/10000/5914/2770/5595/9586/3310/2771/90993/3710/8202/5296/5567/2289/2353/5290/6667/7184/3303/112/107/5580/25984/10488/208/3305/7039/5332/6714/5566	35
hsa04657	IL-17 signaling pathway	25/1495	93/7878	0.038246	3725/3727/3934/3326/5595/5598/9618/4790/23765/5970/2353/6885/7184/4312/7186/2932/5602/3551/8061/7128/5596/51433/4792/23118/8772	25
hsa05418	Fluid shear stress and atheroscler	35/1495	139/7878	0.041558	3725/1843/2817/3326/7132/10000/1003/8878/6383/5598/1514/4790/5970/5296/2353/3554/5290/858/6885/7422/6382/7184/857/5590/9446/	35

	osis				25828/3162/5327/5602/3551/3791/208/4205/7056/6714	
hsa04012	ErbB signaling pathway	23/1495	85/7878	0.042501	3725/9542/1978/817/3265/10000/5595/1027/5063/5296/1399/5290/2002/4609/1026/6777/25/2932/5602/369/208/7039/6714	23
hsa04350	TGF-beta signaling pathway	25/1495	94/7878	0.043043	1387/100532736/1634/4089/4756/4088/5595/64750/7046/2033/7057/4681/7027/656/26585/6667/7040/4609/4092/5518/7042/3398/60436/1030/3625	25
hsa04666	Fc gamma R-mediated phagocytosis	25/1495	94/7878	0.043043	3985/56848/10163/4082/10000/8877/10109/5595/50807/5296/1399/5290/10095/65108/3636/9846/5580/5581/8605/208/8976/4651/81873/55616/1785	25
hsa04668	TNF signaling pathway	29/1495	112/7878	0.04309	3725/602/7132/8837/10000/153090/5595/3726/9586/1435/3659/6376/4790/90993/5970/5296/5606/2353/5290/6885/7186/5602/3551/10488/208/7128/4792/23118/8772	29
hsa05161	Hepatitis B	40/1495	163/7878	0.045036	1387/3725/3265/10000/4089/4088/5595/7046/9586/2033/4790/148022/90993/5970/5296/5606/2353/5290/2002/7040/6885/4609/7529/1026/6777/581/1017/5602/3551/7042/369/4214/10488/208/4775/353376/4792/23118/6714/8772	40
hsa05130	Pathogenic Escherichia coli infection	16/1495	55/7878	0.045456	9475/347733/10381/4691/10109/2534/84617/100506658/10095/7280/81027/25/999/8976/10383/81873	16
hsa04625	C-type lectin receptor signaling pathway	27/1495	104/7878	0.048193	3725/6237/602/8915/3265/10000/5530/5595/5781/23365/3659/4790/3710/5970/5296/5290/22800/4193/5602/5580/3551/208/1540/4775/9261/4792/6714	27

Table S1. Enriched DM genes in KEGG pathways in the T2D dataset. Using the RADAR-detected DM genes in the T2D dataset, we analyzed for enriched KEGG pathways and highlighted a few T2D-related pathways.

Table S2

chr	start	end	name	score	strand	thickStart	thickEnd	itemRgb	blockCount	blockSizes	blockStarts	logFC	p_value
chr15	98648988	98649137	IGF1R	0	+	98649038	98649087	0	1	149	0	-0.663	2.84e-07
chr4	15003223	15003372	CPEB2	0	+	15003273	15003322	0	1	149	0	-1.46	1.05e-06
chr17	80395488	80395637	RNF213	0	+	80395538	80395587	0	1	149	0	-1.27	2.013e-06
chr8	143429196	143429395	MAFA	0	-	143429246	143429345	0	1	199	0	-0.838	5.26e-08
Chr20	382532	388065	TRIB3	0	+	382532	388015	0	2	95,55	0,5429	-0.548	2.37e-0.5

Table S2. Selected DM sites for experimental validation. The table shows the peak information of selected putative DM site from RADAR analysis. The genome coordinate is based on hg38. The shown peak table was extended 50bp towards both upstream and downstream to search for RRACH motif match because the RNA molecules for m_6A-seq was fragmented to ~150 nt but our sequence reads were only 50bp. Consequently, the estimated peak locations could have position shift from the real peak for up to 100 bp. The extension was intended to take this uncertainty into account.

Table S3

Name	Sequence
IGF1R_up	TAG CCA GTA CCG TAG TGC GTG CGC GAC GCA GTT CGC AAG ATC GCC CCG AAG
IGF1R_down	/5Phos/CC GGG TCA CAG GCG AGG CCG GCG AGG GGC CAG AGG CTG AGT CGC TGC AT
TRIB3_up	TAG CCA GTA CCG TAG TGC GTG AGC AAG ATG CAT AAG TAC CAT CCT TGG GAG
TRIB3_down	/5Phos/CT TAG AAA GCT CCC CAG GTT CGA GGC TGG GCA GAG GCT GAG TCG CTG CAT
CPEB2_up	TAG CCA GTA CCG TAG TGC GTG AGC GGC GGA GGC GGC GGC GGC GGC TTC GAG
CPEB2_down	/5Phos/CC GGA GGG TGG GGA AGG TGG GGA GGG CTG ACA GAG GCT GAG TCG CTG CAT
RNF213_up	TAG CCA GTA CCG TAG TGC GTG CCT TCT GAG GCA GAG GTG TAA GCG TTT CAG
RNF213_down	/5Phos/CC CAG ATC GGC TAC AGG GAG TGG CGC TCA GCA GAG GCT GAG TCG CTG CAT
MAFA_up	TAG CCA GTA CCG TAG TGC GTG GGC CTG GTG TCC ACG TCC TGT ACC GCG GAG
MAFA_down	/5Phos/CC GAG CCG AGG CCC CGA GAG GCC TGC GCG ACA GAG GCT GAG TCG CTG CAT
PDX1_up	TAG CCA GTA CCG TAG TGC GTG CTA ATT GAA TAC AAG GAG GCA AAT TCT AAG
PDX1_down	/5Phos/CT GAA CAG AAT ACA GAA AAT TCT GAC AGT CCA GAG GCT GAG TCG CTG CAT
IGF1R_qPCR_F	GCC GCT CAT TCA TTT TGA CT
IGF1R_qPCR_R	CTA GGC GAG GAA AAA CAA GC
TRIB3_qPCR_F	AAC CTT CAG TGC CTT CCA GA
TRIB3_qPCR_R	TGT TGT CAG CTC AAG GAT GC
CPEB2_qPCR_F	TTT CCA CCA AAA GGC TAT GC
CPEB2_qPCR_R	AGC CCT TAA TGG CCT AGG AA
RNF213_qPCR_F	ACA CCT CTG CCT CAG AAG GA
RNF213_qPCR_R	TGA AGG GGC ATT TTT AGC AC
MAFA_qPCR_F	GCG GAG AAC GGT GAT TTC TA
MAFA_qPCR_R	AAG GAA AGG GAG GCT GAG AA
PDX1_qPCR_F	AGC AGT GCA AGA GTC CCT GT
PDX1_qPCR_R	CAC AGC CTC TAC CTC GGA AC

Table S3. Oligo probes sequences and qPCR primer sequences. We designed an up and a down

probe flanking the putative DM m_6A site leaving the m_6A nucleotide as a gap. For each pair of oligo probes, we designed an overhanging universal primer sequence at their 5' and 3' end, respectively. The table shows the sequence of oligo probes we used as well as the qPCR primers we used to quantify gene level variation.
