# Supplementary material for: RADAR: differential analysis of MeRIP-seq data with a random effect model
Source: Genome Biol. 2019 Dec 23;20:294. doi: 10.1186/s13059-019-1915-9 (PMC6927177; doi:10.1186/s13059-019-1915-9)
Supplement: Supplementary file 2 — Additional file 2. Review history. [file 13059_2019_1915_MOESM2_ESM.docx]

Review History

**First round of review**

**Reviewer 1**

**Were you able to assess all statistics in the manuscript, including the appropriateness of statistical tests used?**

No

**Were you able to directly test the methods?**

Yes

**Comments to author:**

MeRIP-seq is a powerful technique to elucidate the m6A modification landscape. In this manuscript, Zhang et al. proposed a novel statistical method, RADAR, to identify the differentially methylated (DM) sites using MeRIP-seq data. The advantages of RADAR are its robust measurement of pre-IP gene expression level and its flexibility in dealing with complex experimental designs. Using simulated count data, the authors demonstrated RADAR outperforms other existing methods. In real MeRIP-seq data, RADAR DM sites advanced functional analyses of m6A.

While the method tackles a significant biological problem and could potentially be a valuable contribution to the epitranscriptomics field, the analyses were not fully rolled out to demonstrate the performance of RADAR, as detailed below.

Major comments:

1. All the performance evaluations in the manuscript were solely based on FDR<10%, while other (arguably more frequently-used) thresholds such as FDR<5% or 1% were not discussed. A more comprehensive analysis would be evaluating the precision using a sliding threshold on all peaks ranked by their significance, similar to Fig. 1 in PMID: 27797762.

2. The simulated sequencing coverage was unclear from the description of simulation analysis. Furthermore, it will be useful to examine the performance of RADAR brought by the robust measurement of pre-IP gene expression on varying sequencing depths.

3. More analyses are needed to fully demonstrate the advantage of RADAR in real data; for example, the motif enrichment and the topological distribution of the m6A DM sites from different callers.

4. Concrete examples of DM sites from RADAR and baseline methods should be shown, besides the current analyses which are mostly summary statistics level. Some exemplar genome browser visualizations of putative DM sites in real data detected by RADAR, but missed by other methods (or vice versa for bogus DM sites) would be very helpful to convince readers to use RADAR.

Minor comments:

5. The information on the number of replicates (Figure S10) is potentially very useful for experimental design. However, the x-axis in a typical ROC curve should be False Positive Rate instead of FDR. Also the authors failed to label the auROC values.

6. page 20 line 26: in computing sensitivity, it was unclear how the simulated read counts (page 20 line 12) were converted to "overlaps with the true sites".

7. page 20 line 35: please justify the choice of covariate effect size.

8. There are several minor grammar errors or typos, such as:
- page 12 line 13-14, specially or specifically?
- page 20 line 4-5, 26324 sites is inconsistent with 26000 sites in the results section (page 9 line 41).
- page 21 line 22: figure S8 should be figure S10.

**Reviewer 2**

**Were you able to assess all statistics in the manuscript, including the appropriateness of statistical tests used?**

Yes: Appropriate

**Were you able to directly test the methods?**

No

**Comments to author:**

Chen and her colleagues develop RADAR, an analytical tool for detecting the differentially methylated loci in MeRIP-seq data. RADAR enables accurate identification of altered methylation sites by accommodating variability of pre-immunoprecipitation expression level and post-immunoprecipitation count using different strategies. The authors show that RADRA could lead to more accurate and reproducible differential methylation analysis results. Below are some of my specific comments:

1.     Y_i (in "Model for DM test" section) is the normalized IP counts. It seems that the authors divide IP by input counts to account for gene expression. Then this Y is not a poisson anymore. So essentially the authors compute the log fold change of normalized IP and input, and then model it through a mixed linear model. The variances of the log fold changes are not properly considered.
2.     The authors compared the performance of RADAR with other existing computational methods. However, there is no experimental validation, which is the key for developing effective computational method.
3.     The manuscript should be better organized and writing could be further improved.

**Authors Response**

**Point-by-point responses to the reviewers’ comments:**

*Response:
We thank both reviewers for their insightful comments and suggestions regarding our manuscript and we appreciate their help for improving this manuscript. We did additional analysis and experiments following the reviewers’ suggestions and integrated those additional data into revised manuscript where appropriate. Below are our point by point responses to reviewers’ comments.*

Reviewer #1: MeRIP-seq is a powerful technique to elucidate the m6A modification landscape. In this manuscript, Zhang et al. proposed a novel statistical method, RADAR, to identify the differentially methylated (DM) sites using MeRIP-seq data. The advantages of RADAR are its robust measurement of pre-IP gene expression level and its flexibility in dealing with complex experimental designs. Using simulated count data, the authors demonstrated RADAR outperforms other existing methods. In real MeRIP-seq data, RADAR DM sites advanced functional analyses of m6A.

While the method tackles a significant biological problem and could potentially be a valuable contribution to the epitranscriptomics field, the analyses were not fully rolled out to demonstrate the performance of RADAR, as detailed below.

Major comments:

1. All the performance evaluations in the manuscript were solely based on FDR<10%, while other (arguably more frequently-used) thresholds such as FDR<5% or 1% were not discussed. A more comprehensive analysis would be evaluating the precision using a sliding threshold on all peaks ranked by their significance, similar to Fig. 1 in PMID: 27797762.

*We thank the reviewer for this great suggestion. We evaluated the performance using a fixed FDR cutoff of 10% simply for easy interpretation in the main text. We agree with the reviewer that evaluations based on sliding FDR cutoffs are more comprehensive and thus we added such analysis in Figure S5 and in the related Section in the main text. We find evaluations at different FDR thresholds have consistent trends with that at 10% FDR cutoff. Therefore, we conclude that RADAR performs better than current alternatives, regardless what FDR cutoff is applied.*


2. The simulated sequencing coverage was unclear from the description of simulation analysis. Furthermore, it will be useful to examine the performance of RADAR brought by the robust measurement of pre-IP gene expression on varying sequencing depths.

*We thank the reviewer for pointing this out. In the Results section (page 7 line 15), we described the simulated sequencing depths (represented as the parameter μ in Equation (1) ), which was based on the mean sequencing depth of the “control” group of the T2D dataset. We have added clarification of this parameter in the Materials and Methods section as well.
We agree with the reviewer that examining the performance of RADAR and other methods with varying sequencing depth is very useful to evaluate the improvement brought by the robust measurement of pre-IP gene expression. Thus, using T2D dataset as an example, we randomly sub-sampled the reads from INPUT samples to scale down the sequencing depth by half. We repeated the analysis on the sub-sampled data and compared the result with that of original data. This analysis showed that RADAR results from the dataset with “shallower” sequencing depth is more consistent with the results from the dataset with “deeper” sequencing depth, compared to other methods as shown in Figure S9, demonstrating robustness to sequence depths offered by RADAR. We have added a paragraph describing results of this analysis in the Results Section (page 11 line 18).*


3. More analyses are needed to fully demonstrate the advantage of RADAR in real data; for example, the motif enrichment and the topological distribution of the m6A DM sites from different callers.

*We thank the reviewer for these two great suggestions. The main aim of RADAR is to detect differentially methylated (DM) sites instead of methylation sites detection by peak calling although differential sites detected by RADAR are also expected to be methylated sites. To demonstrate that RADAR-detected differentially m6A methylated sites are indeed consistent with known characteristics of m6A sites, we performed de-novo motif search on RADAR-detected DM sites in real data. We showed that RADAR-detected DM sites are enriched for known m6A consensus motif (RRACH) and plotted the topological distribution of DM sites in Figure S10.
We’ve also compared the motif enrichment analyses of the DM sites from different methods. We found that the p-value of motif enrichment are mainly correlated to the number of DM sites detected (e.g. in ovarian cancer: exomePeak—12072 DM sites, motif p-value=1e-527; RADAR—2266 DM sites, p-value=1e-95; MeTDiff—585 DM sites, p-value=1e-30). We think this is because DM sites detected by all methods are mostly true m6A sites, thus more m6A sites led to better p-value in motif discovery. However, whether an m6A site is true DM site is not related to the consensus motif. The large number of DM sites detected by exomePeak could be mostly true m6A site but large portion of them are not truly differentially methylated. Thus, we think motif enrichment and topological distribution of m6A DM site provide useful information but is not suitable for assessing the accuracy of differential methylation sites.*

4. Concrete examples of DM sites from RADAR and baseline methods should be shown, besides the current analyses which are mostly summary statistics level. Some exemplar genome browser visualizations of putative DM sites in real data detected by RADAR, but missed by other methods (or vice versa for bogus DM sites) would be very helpful to convince readers to use RADAR.

*We agree with the reviewer that concrete examples with visualization of raw data are important demonstration of RADAR’s performance. We added examples of DM sites detected only by RADAR as well as bogus DM sites that are likely false discovery driven by a few outliers. We showed coverage plot for individual samples in Figure S7.*

Minor comments:
5. The information on the number of replicates (Figure S10) is potentially very useful for experimental design. However, the x-axis in a typical ROC curve should be False Positive Rate instead of FDR. Also the authors failed to label the auROC values.

*We corrected the x-axis for this figure and added auROC values in the figure, which is now Figure S13.*


6. page 20 line 26: in computing sensitivity, it was unclear how the simulated read counts (page 20 line 12) were converted to "overlaps with the true sites".

*We clarified this procedure in the Method s¬¬¬¬¬ection. Briefly, we simulated read counts according to Equation (1), where a fold change was introduced to one group of samples on the methylation levels of pre-defined true DM sites (20% of all simulated sites). We applied the benchmarked methods on the simulated read counts. We then checked if pre-defined true sites are predicted to be DM site at varying FDR cutoffs.*

7. page 20 line 35: please justify the choice of covariate effect size.

*We have added justification for our choice of covariate effect size. Our choice is motivated by the covariate with the largest effect size in the T2D dataset, which ranges from 0 to 4. We chose a median of the effect size range— 2— to represent a moderately challenging scenario we could encountered in real data.*

8. There are several minor grammar errors or typos, such as:
- page 12 line 13-14, specially or specifically?
- page 20 line 4-5, 26324 sites is inconsistent with 26000 sites in the results section (page 9 line 41).
- page 21 line 22: figure S8 should be figure S10.

*We thank the reviewer to point out these errors. We have corrected these errors.*

Reviewer #2: Chen and her colleagues develop RADAR, an analytical tool for detecting the differentially methylated loci in MeRIP-seq data. RADAR enables accurate identification of altered methylation sites by accommodating variability of pre-immunoprecipitation expression level and post-immunoprecipitation count using different strategies. The authors show that RADRA could lead to more accurate and reproducible differential methylation analysis results. Below are some of my specific comments:

1. Y_i (in "Model for DM test" section) is the normalized IP counts. It seems that the authors divide IP by input counts to account for gene expression. Then this Y is not a poisson anymore. So essentially the authors compute the log fold change of normalized IP and input, and then model it through a mixed linear model. The variances of the log fold changes are not properly considered.
*Y_i in our model is a pre-processed IP counts. We first each normalized INPUT and IP counts to account for library size and IP efficiency variations. We then divide the IP counts by a gene-specific size factor (this size factor is computed by rescaling the normalized INPUT counts toward 1). Therefore, the Y_i is still at its original scale after dividing the rescaled INPUT counts.
We used the gene-level counts as the estimate of pre-IP RNA expression level to mitigate the dispersion by increasing the number of reads and simultaneously diminishing the effects of sequence characteristics within a gene. The variance of normalized read counts is captured through a random effect term in a generalized linear model.*

2. The authors compared the performance of RADAR with other existing computational methods. However, there is no experimental validation, which is the key for developing effective computational method.

*We fully agree with the reviewer that experimental validation provides support to the result obtained by computational methods. Indeed, one of the real datasets (T2D) analyzed in this paper led to the discovery of connection between m6A variation and T2D biology. As discussed in our manuscript, the identified key DM genes and their associated pathways were further studied and verified to contribute to beta-cell function and T2D biology. Results are presented in detail in another manuscript we recently published at Nature Metabolism [1]. The m6A-modulated pathway validated in the T2D paper was also shown in Figure S12. This actually provided an orthogonal evidence to support that the DM sites we identified using RADAR are likely true and biologically important.
In addition, we performed additional experiments to directly validate the result obtained by RADAR. Validation of the DM sites are challenging because there is no gold standard for high-throughput m6A quantification, to the best of our knowledge. However, a low-throughput m6A quantitative analysis method (termed SELECT) published recently can validate our putative DM sites in an antibody pulldown-independent manner. Thus, we selected 6 DM sites from the T2D dataset for experiment validation. 2 sites were selected from top DM sites that were only detected by RADAR but not by other methods. 4 sites were selected from key genes that were found to contribute to T2D biology in our previous study. We showed 5 out of 6 DM sites we tested showed consistent direction of change with RADAR result. 2 of these 5 validated sites were statistically significant by SELECT method. 1 out 6 tested sites didn’t show changes as predicted by RADAR analysis. We note that the power of SELECT analysis is limited due to small sample size, which is limited by the availability of remaining RNA sample from earlier sequencing (These are all patient samples and are rare). Thus, most tested sites showing consistent direction of methylation alteration suggests that RADAR detected DM sites are mostly true positives. We added results (Fig. 6) and experimental procedures in the Result and Method sections, respectively.*
3. The manuscript should be better organized and writing could be further improved.

*We have revised our manuscript to improve clarity.


Reference
1. De Jesus DF, Zhang Z, Kahraman S, Brown NK, Chen M, Hu J, Gupta MK, He C, Kulkarni RN: m6A mRNA methylation regulates human β-cell biology in physiological states and in type 2 diabetes. Nature Metabolism 2019.*

**Second round of review**

**Reviewer 1**

The authors have addressed my concerns satisfactorily. The revised manuscript demonstrated the usefulness of their method from both statistical and biological perspectives.

**Reviewer 2**

The authors have addressed my concerns.
